# Association between age and muscle function, architecture, and composition in long-distance master runners: a cross-sectional study

**DOI:** 10.1590/1414-431X2022e12383

**Published:** 2022-11-11

**Authors:** J. Teixeira, A.G. Brauer, A.E. Lima-Silva, P.C.B. Bento

**Affiliations:** 1 Departamento de Educação Física, Universidade Federal do Paraná, Curitiba, PR, Brasil; 2 Centro de Estudos do Comportamento Motor, Programa de Pós-Graduação em Educação Física, Universidade Federal do Paraná, Curitiba, PR, Brasil; 3 Departamento de Educação Física, Unibrasil Centro Universitário, Curitiba, PR, Brasil; 4 Grupo de Pesquisa em Performance Humana, Departamento Acadêmico de Educação Física, Universidade Tecnológica Federal do Paraná, Curitiba, PR, Brasil

**Keywords:** Master runners, Master athletes, Musculoskeletal system, Endurance runners, Aging

## Abstract

The aim of this study was to describe the muscle function, architecture, and composition of long-distance master runners, and verify the association between age and these variables. Additionally, different clusters of runners were compared based on age and training variables. Forty male runners (≥50 years) reported their training routine and had their muscle function evaluated through maximum knee extensor isometric peak torque (PT) assessed with an isokinetic dynamometer. The cross-sectional area (CSA), pennation angle (PA), fascicle length (FL), muscle thickness (MT), and echo intensity (EI) were evaluated through ultrasound (muscle architecture and composition). The participants were 58.7±6.2 years old and had been training for 18.4±10.3 years, 4 sessions/week with 298.8±164.7 min/week of training. The absolute torque was 226.92±63.44 N·m, and the specific torque (PT/CSA) was 7.29±3.78 N·m/cm^2^. Regarding muscle architecture, the phase angle was 17.34±4°, the fascicle angle 6.78±1.04 cm, muscle thickness 2.93±0.56 cm, and the cross-sectional area 21.24±5.88 cm^2^. Concerning muscle composition, the master runners showed echo intensity values of 62.05±11.68 AU. The analysis demonstrated a weak and negative association between age and some muscle architecture variables (CSA and MT) and muscle function (PT). No association was verified between age and muscle composition (EI). Age partially explained CSA, MT, and muscle function changes (13, 11, and 14%, respectively). Participants' high level of physical training might have contributed to the low association between these variables and the lack of association with muscle composition.

## Introduction

Muscle mass decreases approximately 3-8% per decade after 30 years, and this rate accelerates after 60 years (1). Also, the rate of fat or non-contractile tissue infiltration into muscle compartments (muscle composition) is approximately 18% per year (2), and it is associated with low muscle strength and poor physical performance (3). In addition, poor muscle function is associated with the combination of a reduction in muscle cross-sectional area, pennation angle, fascicle length, and muscle thickness (4,5). These age-related alterations can be accentuated due to reduced physical activity level, which usually happens with aging (4).

However, master athletes are part of the older population that remains very physically active. These athletes have high levels of physical training, systematically train to compete in organized sport events, and are considered a biological model for understanding a successful aging process (5). Despite the reduced volume and intensity of training with advancing age, which might contribute to the increase in the rate of decline of some physical and physiological abilities, the level of physical activity of these athletes remains high compared to non-athletes of the same age (4).

Most studies with long-distance master runners have shown the adaptations and changes that can occur with aging in cardiorespiratory fitness and metabolic variables (5). Others have analyzed the effects of strength training in muscle function, architecture, and composition of the runners (6). Few studies, however, have investigated the effects or association of specific endurance run training and age-related alteration in muscle function, composition, and architecture in long-distance master runners, and the results are inconclusive. Some studies found that running training might mitigate age-related alterations of the neuromuscular system, such as loss of motor units and size and distribution of Type I and Type II fibers (7). On the other hand, other studies demonstrated that endurance training alone might not preserve age-related muscle function and architecture, and supplementary training is required (8,9). Thus, the possible protective effect of long-distance running training on age-related alteration in muscle function, architecture, and composition is not well established.

Therefore, this study aimed to describe the muscle function, architecture, and composition of long-distance master runners, and verify the association between age and these variables. Additionally, the different clusters of runners were compared based on age and training variables.

## Material and Methods

### Participants

This study had a cross-sectional design and was conducted from June to December, 2018. Participants were male runners who participated in the local running events promoted by the Municipal Sports Secretary of the city of Curitiba in Paraná, Brazil. The mean number of participants in these events was 1649 men, of which 21.2% were aged 50 or older. The sample size was calculated using the Epinfo calculator developed by the Centers for Disease Control and Prevention (USA) (10). The following parameters were used: 21.2% of 1649 event participants indicating an N=350 subjects, anticipated frequency of 50%, confidence level of 10%, design effect of 1.0. A total sample size of 40 participants resulted in a statistical power of 80%.

Recreational master runners who were 50 years of age or older, had been training for a minimum of 5 years, had a weekly running volume of ≥15 km, and participated in official running events were recruited. Also, they could not present recent orthopedic injuries (muscles, tendons, joints, ligaments, and bones) that limited the performance of the strength test and ultrasound image.

Furthermore, the athlete's index was calculated using the Age-Graded Calculator program (http://www.mastersathletics.net/index.php?id=2595), utilizing age and the current personal updated record in each event compared to world records (10 and 42.190 km). The reference for classification was the local (≤70%), regional (>70%), national (>80%), international (>90%), or world record level (100%).

### Procedures

Volunteers were previously contacted by telephone to verify that they met the inclusion criteria. After that, they visited the laboratory once, received details about the aims and protocols involved in the study, and signed an informed consent form. Then, the evaluations were carried out in three steps: i) answering a questionnaire (training routine and personal record in their current age category) and anthropometric measurements; ii) assessment of muscle composition and architecture; iii) muscle function evaluation. The research was approved by the Federal University of Paraná (UFPR) Ethics Committee (Approval IRB: 2.259.186).

#### Questionnaire and anthropometric assessment

Participants answered a questionnaire about the training routine such as training frequency, weekly training distance, duration of the session, distance races, and personal record. Then, they underwent anthropometric assessment (height and body mass).

#### Assessment of muscle composition and architecture

Muscle measurement was conducted using ultrasound (Konica Minolta^®^, model Sonimage HS, Japan). The vastus lateralis (VL) muscle was evaluated because it can be representative of the quadriceps femoris. In addition, all muscles of the quadriceps group have similar age-related atrophy (11). The choice of this muscle was also due to its importance for running, especially in the braking phase (12). The VL of the right thigh was assessed using ultrasound, with a transducer 5 cm long by 2 cm wide, at a frequency of 11 MHz. The measurements collected were muscle architecture (cross-sectional area [CSA], pennation angle [PA], fascicle length [FL], muscle thickness [MT]), and muscle composition by echo intensity (EI).

**Figure 1:**
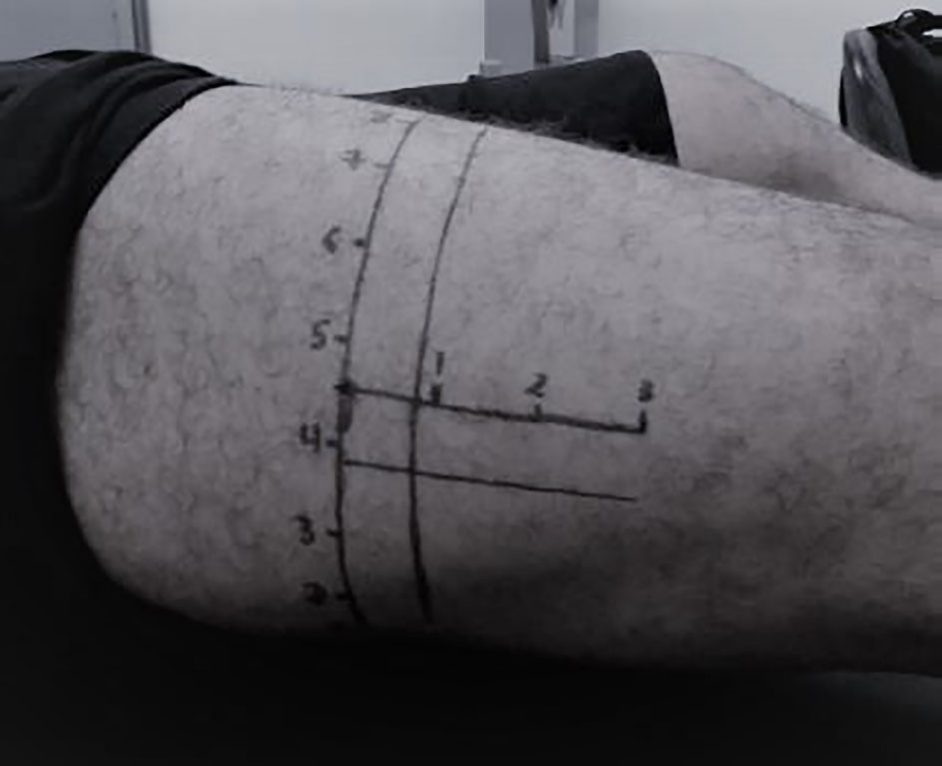
Marking template for ultrasound assessment.

The subjects remained at rest for 15 min in a supine position with the leg extended and relaxed to accommodate body fluids (13). The VL was identified between the greater trochanter and the lateral epicondyle of the femur (midpoint) of the right leg. After being identified, axial sections were marked on the skin at 30 mm intervals from that point, as proposed in the literature (14) (Figure 1). Then, a conductive water-based gel was applied over the site.

The transducer was aligned perpendicular to the VL muscle and moved from a lateral to a central position along the pre-demarcated section, oriented in the axial plane, to measure CSA. For FL, PA, MT, and EI measurements, the transducer was aligned longitudinally to the muscle. The transducer was applied without pressure or skin compression during the scan. After a satisfactory monitor image with visible and clean muscle fascia was found, the image was archived for further analysis (15).

**Figure 2:**
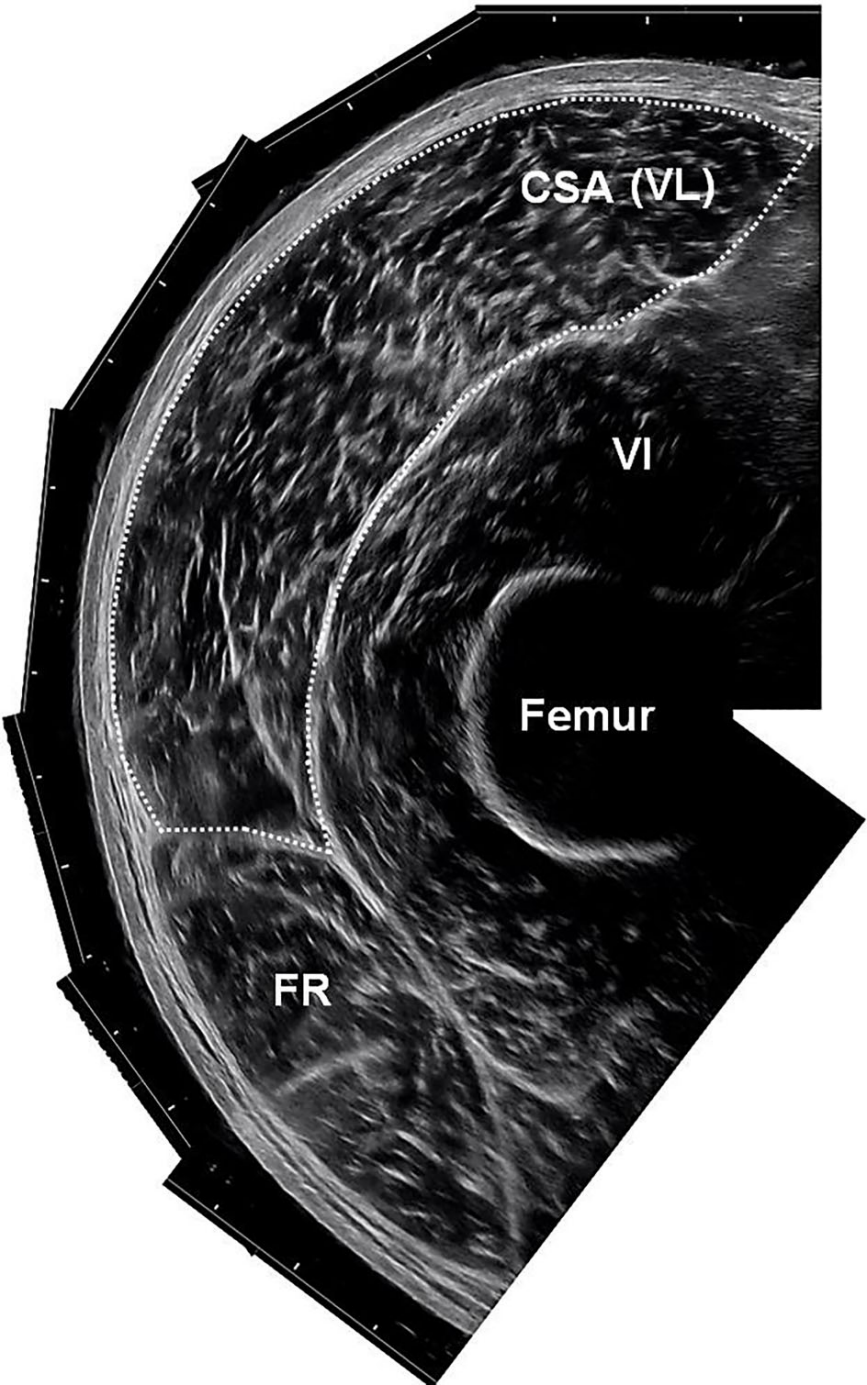
Reconstitution of the ultrasound image of the vastus lateralis (VL) and demarcation of the cross-sectional area (CSA). VI: vastus intermedius; FR: rectus femoris.

The images were sequentially opened in PowerPoint (Microsoft, USA). Then, each image was manually rotated until the reconstruction of the entire fascia of the VL (14). The CSA of the VL was measured using computerized planimetry, using the computer mouse. ImageJ software (version 1.5; NIH, USA) was used to measure, in millimeters, the linear distance between the fat and muscle and between the muscle and bone. The CSA was determined by the contour of the VL (Figure 2).

**Figure 3:**
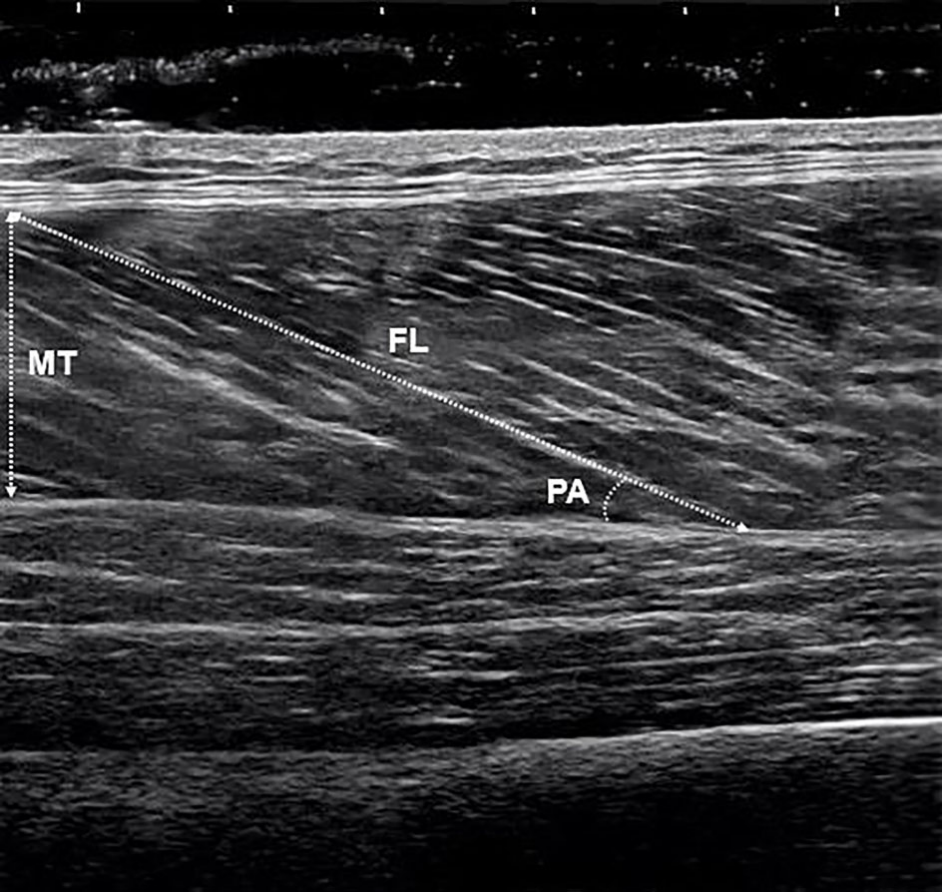
Architectural measurements of the vastus lateralis (VL) in the ultrasound image. PA: pennation angle, FL: fascicle length, MT: muscle thickness.

The PA was determined by the angle formed between the muscle fascicles and the internal aponeurosis, whose orientation coincides with the muscle traction line. To measure the FL, we determined the linear distance between the origin of the fascicle in the internal aponeurosis and the respective insertion in the external aponeurosis (epimysium). MT is an indirect parameter of the CSA and muscle volume and was determined by the perpendicular distance between the fat and muscle and between muscle and bone (Figure 3).

The EI was measured by grayscale analysis of the infiltration of fat and non-contractile elements into the muscle, using the standard histogram function in ImageJ. The pixels within the area of interest were processed using the fast Fourier transform, resulting in a distribution of 256 shades of gray, with 0=black and 256=white. Thus, lighter pixels (hyperechoic) indicate infiltrated fat and non-contractile elements in the muscle (16). The EI was calculated as the median of the values within the CSA and is reported in arbitrary units (AU).

A single experienced evaluator performed all measurements. For reliability and reproducibility of measurements, a pilot study with ten volunteers was carried out on two different days as recommended (17). The coefficients of variation (CV), intraclass correlation coefficient (ICC), and the standard error (SE) of measurements were calculated. Values of CV, ICC, and SE for cross-sectional areas were 0.11, 0.97, and 0.28, for fascicule lengths were 0.12, 0.89, and 0.29, for pennation angle were 0.13, 0.94, and 0.69, and for muscle thickness were 0.09, 0.81, and 0.08, respectively.

#### Muscle function assessment

The right knee extensors' maximal isometric voluntary contraction (MIVC) was measured using an isokinetic dynamometer (Biodex System 3, Biodex Medical System, USA) calibrated following the manufacturer specifications. For the positioning of the Biodex, we assumed the device manufacturer's guidelines, considered the gold standard for assessing the CVIM of knee extensors (18).

Participants were placed in a seated position, and the dynamometer rotation axis of the right knee joint (a line traversing the femoral epicondyles) was determined. The chair's back was set at 90°, and the lever arm was adjusted and fixed 2 cm above the malleoli of the ankle. The regions of the trunk, pelvis, and thigh were stabilized with straps.

A warm-up and familiarization period with the equipment was conducted. Subjects performed one set with four repetitions of MIVC of the knee extensors, with each repetition lasting five seconds and with an interval of 60 s for each repetition (19). Muscle contraction intensity increased progressively with repetitions (approximately 25, 50, 75, and 100% of maximum strength). After warm-up, there was a 2-3 min rest time before starting the test. The test protocol included three repetitions of MIVC with a 120-s interval between repetitions. The subjects were verbally encouraged to perform the strongest and fastest contraction possible during 5 s. During all MIVCs, the subjects visualized their torque curves on the dynamometer monitor as visual feedback (20). The highest peak torque (PT) value achieved in the three maximum efforts was taken as the MIVC, and the ratio of PT and CSA was calculated to obtain the specific torque (N·m/cm^2^) (6,21).

### Statistical analysis

Data normality was tested using the Kolmogorov-Smirnov test, and Pearson's correlation and Spearman's correlation tests were used for parametric and non-parametric data, respectively, to investigate the possible associations between age and the analyzed variables (muscle function, muscle architecture, and muscle composition). When correlations were detected, we applied an additional test to verify the correlation between muscle variables, weekly training volume, and years of training.

The r correlation coefficient was determined to check the magnitude of the observed associations, considering r<0.30 very weak if any correlation, from 0.30 to 0.50 weak correlation, from >0.50 to 0.70 moderate correlation, from >0.70 to 0.90 strong correlation, and r>0.90 very strong correlation (22).

In addition, a clustering procedure was carried out based on age, years of training, and weekly training volume. K-means clustering is a technique used to find an optimal number of centers (K) that relate to the data set in such a way that the distance between the centers and the data points is minimized (23). This forms groups based on any number of variables where objects in the same group are as similar as possible, and objects of different groups are dissimilar as possible. The cluster analysis was validated via the silhouette coefficient, presenting a value of 0.53 for Cluster 1, 0.46 for Cluster 2, 0.10 for Cluster 3, and average silhouette measure of cohesion and separation (0.35).

After this analysis, we identified three groups: G1 (n=22): 54.5±2.6 years old, 13±6.7 years of training, 242.9±104 min/week; G2 (n=12): 65.0±3.4 years old, 19.8±9.3 years of training, 245.8±73.4 min/week; G3 (n=6): 61.0±8.5 years old, 34.8±2.5 years of training, 610±132.3 min/week. To compare the musculoskeletal variables between groups, a general linear model was applied (unbalanced groups). The significance level was set at P<0.05, and all statistical analyses were performed using SPSS software (version 20; IBM Corp., USA).

## Results

The participants' main characteristics concerning age, anthropometric data, training routines, and musculoskeletal variables are shown in Table 1. The participants were considered to be at regional level of performance and presented 69.5 and 68.4% of the age-group world record for 10 kilometers and marathon, respectively. They had been training for 18 years, in 4 weekly sessions, and for approximately 300 min per week. Some participants practiced complementary training as follows: weight lift (10 participants), pilates (8 participants), and swimming (5 participants). In addition, other forms of training such as spinning (1 participant), cycling (3 participants), ballet (1 participant), and functional training (3 participants) were also cited.

**Table 1:** Participants' characteristics and muscle variables (means±SD, and range).

Variable	Values
Age (years)	58.70±6.28 (50-71)
Body mass (kg)	75.55±12.45 (60-98)
Height (cm)	174.78±6.49 (168-191)
Body mass index (kg/m^2^)	24.71±1.13 (18.9-37.9)
Practice time (years)	18.40±10.38 (6-37)
Training sessions/week (days)	4.08±1.68 (2-12)
Training volume/week (min)	298.8±164.7 (120-720)
Peak torque (N·m)	226.92±63.44 (129.13-400.36)
Peak torque/VL CSA (N·m/cm^2^)	7.29±3.78 (6.94-18.35)
Cross-sectional area (cm^2^)	21.24±5.88 (13.73-36.43)
Fascicle length (cm)	6.78±1.04 (4.66-8.89)
Muscle thickness (cm)	2.93±0.56 (1.33-3.22)
Pennation angle (°)	17.34±4.00 (6.95-26.68)
Echo intensity (AU)	62.05±11.68 (35-89)

VL: vastus lateralis; CSA: cross-sectional area; AU: arbitrary units.

Regarding muscle function assessed by MIVC, the absolute torque was 226.92±63.44 N·m, and specific torque (PT/CSA) was 7.29±3.78 N·m/cm^2^. For muscle architecture, the PA was 17.34±4°, the FL 6.78±1.04 cm, the MT 2.93±0.56 cm, and the CSA 21.24±5.88 cm^2^. Concerning muscle composition, the master runners showed EI values of 62.05±11.68 AU.

**Figure 4:**
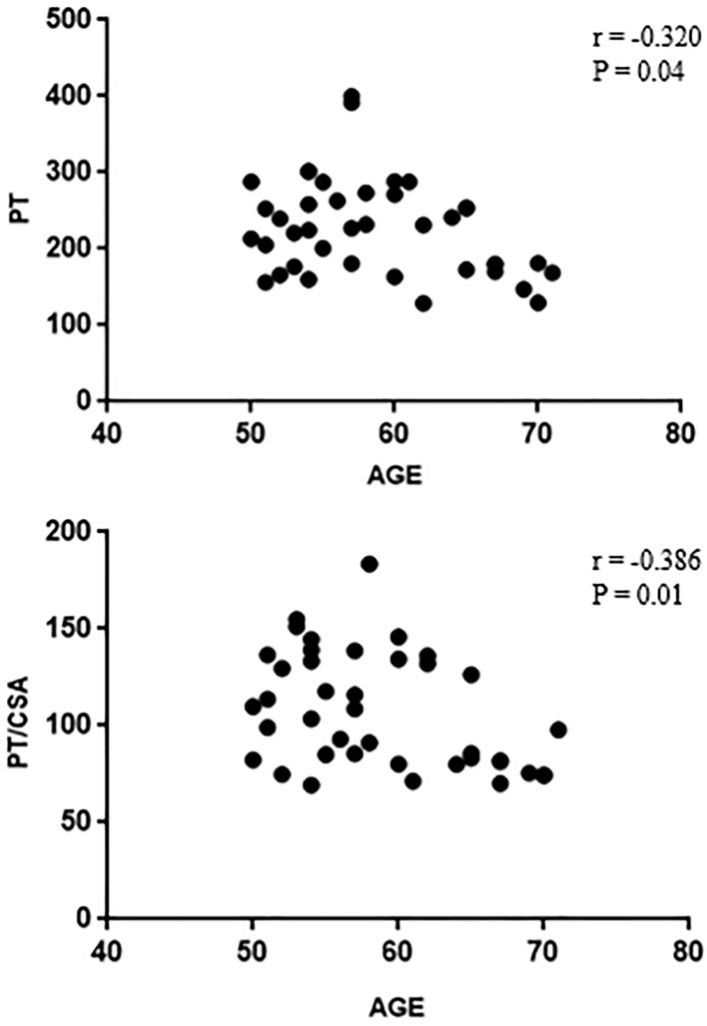
Correlation between age and muscle function. PT: peak torque; CSA: cross-sectional area.

A weak and negative correlation was found between age and muscle function for absolute PT (r=-0.320, R^2^=0.10, P=0.04) and specific torque (torque/CSA) (r=-0.386, R^2^= 0.14, P=0.01). Thus, age explained 14% of the variation in the muscle function of the runners. The correlations between age and PT and age and PT/CSA are shown in Figure 4.

**Figure 5:**
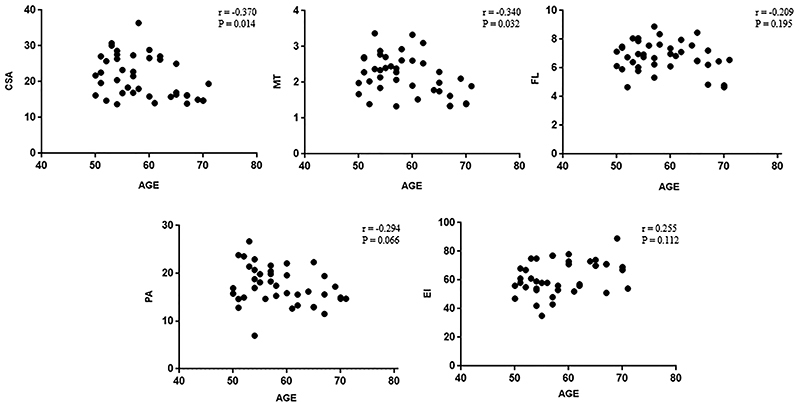
Correlation between age and muscle architecture and composition. CSA: cross-sectional area; MT: muscle thickness; FL: fascicle length; PA: pennation angle; EI: echo intensity.

Based on the results of the correlation between age and muscle architecture, a weak and negative correlation was determined between age and CSA (r=-0.370, R^2^=0.13, P=0.01) and age and MT (r=-0.340; R^2^=0.11, P=0.03). Age explained only 13% and 11% of the variation observed in the muscle architecture variables of the runners. There was no significant correlation between age and PA (r=-0.294, R^2^=0.08, P=0.06) and age and FL (r=-0.209, R^2^=0.04, P=0.19). Similarly, no significant correlation was found between age and EI (r=0.255, R^2^=0.06, P=0.11). The results of correlations between age and muscle architecture and composition variables are shown in Figure 5.

Considering that only a small part of the variation in the musculoskeletal variables can be explained by age, a complementary analysis was performed to verify if there was a correlation between CSA, MT, and specific peak torque and weekly training load and training duration (years).

No significant correlation was observed between training load and CSA (r=-0.105, R^2^=0.011%, P=0.520), MT (r=-0.170, R^2^=0.02%, P=0.295), and specific torque (r=-0.248, R^2^=0.06%, P=0.123). Similarly, no significant correlation was found between training duration and CSA (r=0.000, R^2^=0%, P=0.998), MT (r=-0.020, R^2^=0.0004, P=0.904), and specific torque (r=-0.001, R^2^=0%, P=0.995).

The comparison between three groups (clusters) is shown in Table 2. Fascicle length was found to be different between groups. The group formed by middle-aged runners (G1), who had a low weekly load and for fewer years of training (∼13 years) or fewer years of practice, showed greater fascicle length than the other two groups of older runners with more training time per week and more years of practice. In addition, a shorter fascicle length was observed in the runners who had been training for more than 34 years and with a weekly training load almost three times greater than the other groups (G3). Also, CSA was 4 cm^2^ greater in (G1) compared to G3, although the difference was not statistically significant (P=0.09). There were no differences between groups for the MT and EI.

**Table 2:** Muscle variables according to clustering by age, years of training, and weekly min of training (means±SE and range).

Variable	Groups	Estimated marginal means		95%CI		Contrasts	Estimate
		Estimate	SE	Lower	Upper		
CSA	1	23.252	1.261	20.699	25.805	1 *vs* 2	-2.006
	2	21.246	0.886	19.452	23.040	1 *vs* 3	-4.013
	3	19.239	1.261	16.686	21.793	2 *vs* 3	-2.006
FL	1	7.176^ab^	0.220	6.731	7.621	1 *vs* 2	-0.396
	2	6.781^b^	0.154	6.468	7.093	1 *vs* 3	-0.792
	3	6.385	0.220	5.940	6.830	2 *vs* 3	-0.396
MT	1	2.373	0.121	2.128	2.617	1 *vs* 2	-0.184
	2	2.189	0.085	2.017	2.360	1 *vs* 3	-0.368
	3	2.005	0.121	1.760	2.249	2 *vs* 3	-0.184
PA	1	18.752	0.855	17.022	20.482	1 *vs* 2	-1.407
	2	17.344	0.600	16.129	18.560	1 *vs* 3	-2.814
	3	15.937	0.855	14.207	17.667	2 *vs* 3	-1.407
EI	1	18.752	2.628	54.807	65.448	1 *vs* 2	1.923
	2	62.050	1.847	58.312	65.788	1 *vs* 3	3.845
	3	63.973	2.628	58.652	69.293	2 *vs* 3	1.923

CSA: Cross-sectional area; FL: fascicle length; MT: muscle thickness; PA: pennation angle; EI: echo intensity. ^a^P<0.05 group 1 compared to group 2; ^b^P<0.05 group 1 compared to group 3, ^c^P<0.05 group 2 compared to group 3. P-values were adjusted using Tukey adjustment.

## Discussion

To the best of our knowledge, this is the first study to investigate the correlation between age and muscle function, muscle architecture, and muscle composition of long-distance master runners. The main finding was that even in a group of master runners, there is a weak and negative association between age and some muscle architecture variables (CSA and MT) and muscle function. However, it is essential to highlight that this association explained a small part of the variation. Furthermore, the complementary analyses failed to demonstrate any correlation between the muscle-skeletal morphological variables and training load (years of training and weekly mileage).

During the normal aging process, in the absence of disease and intense physical training, changes in the musculoskeletal system have been reported. Studies have indicated age-related declines in muscle variables, such as reduced muscle function due to changes in muscle architecture and infiltration of non-contractile tissue in the muscle, changes in the composition and capillarization of muscle fibers, and reduction in muscle mass (4,5).

Studies that compared master athletes who train and compete in long-distance trials with non-active older people found conflicting results. For example, higher maximal strength and a greater muscle fiber diameter were found in master athletes (24). On the other hand, other studies have found similar values for muscle size, strength, and CSA of the muscle fibers among master athletes and non-active older adults (25,26). These conflicting results may partially be explained by factors such as participants’ age, level of performance, training routine, and lifelong training time (27). In addition, changes in muscle strength may be associated with structural and functional changes in skeletal muscle (28), such as reduced CSA, PA, and FL (29).

In one study, a group of young adult men was compared with older adult men, both physically active. Although the older group was physically active, they presented a lower CSA of the VL muscle than the young group (27). Thus, the authors state that even in natural aging, neuronal motor losses occur, and instability in neuromuscular transmission and other factors can reduce the CSA.

In the present study, the association between age and muscle architecture and function was weak and explained only 13% of changes in CSA and 11% in MT, which may be due to the high level of physical activity of the participants.

The PA has a direct relationship with force produced (30), where the greater the angle, the greater the force transmitted to the tendon (31). Also, the PA is directly related to the FL since the greater the FL, the smaller the PA resulting in a greater magnitude of contraction velocity (32). As the muscle contracts, there is a shortening of the fibers along the tendon. Thus, the higher the PA, the shorter the fiber spacing, suggesting that the fibers are stretched and operate closer to their ideal length (28,33,34). In the present study, no correlation was observed between age and PA and FL, which may reflect the chronic training of these athletes.

Studies have shown that the aging process promotes the infiltration of fat and non-contractile elements into the muscle, resulting in decreased muscle function (35). The torque capacity per unit of muscle mass (specific torque) is reduced when there is a great amount of intramuscular fat. Although a correlation between muscle strength and the EI is suggested, no change was observed in this variable after muscle strength training, even though an increase in strength was observed (1). Another study compared the EI of master athletes of power and endurance sports, including long-distance runners and sedentary older adults, all with an average age of around 70. It was found that the EI was lower in both groups of athletes compared to sedentary ones, suggesting, according to the authors, a protection due to lifelong training time (26).

Although the master runners were not compared with their sedentary peers in the present study, no correlation was observed between age and EI, an indirect measure of fat infiltration into the muscle (26). In the present study, there was a weak and negative correlation between specific torque and age, to which the lack of correlation between age and EI may have contributed. Therefore, the muscle infiltration of fat and non-contractile elements in this group of athletes is not enough to explain muscle function.

Finally, regarding cluster analysis, the groups of runners with longer lifelong training (19 and 39 years old) had shorter fascicle length than the younger group (54 years old) with shorter training time (13 years) and less weekly training time. There are some possibilities to explain these results. First, the two groups with shorter FL were older than the G1, and an age-related shortening in FL was reported previously in untrained older adults (36). Second, the G1 presented a greater VL cross-sectional area than the other groups (although not statistically significant), especially compared to the G3 (4 cm^2^), and the greater the CSA the greater the FL (31). Finally, the shorter FL observed in the group with more experience (years) and weekly training time (G3) may be an adaptation to life-long endurance training. It was demonstrated previously that older endurance runners had shorter soleus fascicles than young and older untrained adults. Shorter fascicles may reduce the energy cost of force production due to lower activated muscle volume per unit of force output compared with longer fascicles (37). However, considering that muscle architecture may vary according to muscle group and sport, we must be cautious with this affirmation (38).

A major limitation of this study is the lack of a comparative group of young or sedentary older adults or both. Another limitation was that only the right leg was assessed instead of the dominant leg due to the equipment’s setup (Biodex and ultrasound). However, some studies have reported no significant differences between the dominant and non-dominant leg (39), and there is no consensus in the literature (40). Also, this was a cross-sectional study, so it is not possible to establish a cause-and-effect relationship.

In conclusion, a weak and negative correlation was observed between some muscle variables and age, specifically normalized torque, cross-sectional area, and muscle thickness. No association was verified between age and muscle composition (EI). These results showed that age partially explained CSA, MT, and muscle function changes (13, 11, and 14%, respectively) and that the participants’ high level of physical training might have contributed to the weak association between these variables and the lack of association with muscle composition. Regarding the clustering procedure, the groups of runners with the longest time of practice (19 and 39 years) presented shorter FL compared to the less experienced group (13 years). A comparison with non-athletes in the same age group with different physical activity levels would help to better understand the role of endurance running training on the musculoskeletal system during the aging process. Also, longitudinal studies are required to determine whether lifelong endurance running training may protect or postpone age-related musculoskeletal system alterations of master running athletes.
